# Updating the “cost factor” in the William fine risk assessment method following economic power: A case study of selected production industries in Iran

**DOI:** 10.1016/j.heliyon.2025.e42405

**Published:** 2025-01-31

**Authors:** Bafrin Moloudpourfard, Morteza Tahamipour Zarandi, Mostafa Pouyakian

**Affiliations:** a Department of Occupational Health Engineering, School of Public Health and Allied Medical Sciences, Iranshahr University of Medical Sciences, Iranshahr, Iran; b Department of Economics, Faculty of Economics and Political Science, Shahid Beheshti University, Tehran, Iran; c Department of Occupational Health and Safety Engineering, School of Public Health and Safety, Shahid Beheshti University of Medical Sciences, Tehran, Iran

**Keywords:** Control measures, Cost factor, Risk assessment, Safety cost, William fine

## Abstract

The William Fine approach is among the limited number of methods, introducing a metric referred to as the J index and discussing safety costs to evaluate control measures. Therefore, this study aimed to update the “cost factor” in the William Fine method in proportion to the economic power in selected production industries in Iran. Based on the International Standard Industrial Classification (ISIC), three selected production industries were the automotive, pharmaceutical, and food industries. The Delphi technique was used to achieve the values of safety costs in economic sectors. Due to high exchange rate fluctuations and the possibility of using the cost invoice table over time and the producer price index, the correction factor was calculated. Furthermore, the fuzzy logic method determined the cost factor of William Fine's method. The gap between the minimum and maximum costs is significantly broader in the automotive industry compared to the pharmaceutical and food industries. The cost factor correction for the automotive industry stands at 1.144, while it is 1.222 for pharmaceuticals and 1.141 for the food industry. These figures are expected to be generalized in the coming years. The present study revealed that due to the impossibility of accurate access to data related to cost performance and safety investment in industries, the Delphi method could be acceptable to achieve the range of safety costs in industries. Updating this table has enabled selected industries to achieve more accurate control costs or risk aversion estimates.

## 1 Introduction

Risk identification and risk assessment are fundamental to adopting a proactive safety approach [1]. Risk assessment constitutes an industry's crucial targeted risk control methodology [2]. Over the past few decades, numerous safety risk assessment techniques have been proposed and implemented in a multitude of studies [3]. Typically, these techniques rely on incorporating two key parameters: Severity and probability. In select instances, an additional parameter, such as the rate of detection or contact, is utilized to evaluate identified hazards [4]. The MIL-STD 882E method [5], including two-parametric methods, as well as William Fine and RPN FMEA methods [6,7], serve as examples of three-parametric methods for risk assessment. Within each method, assessments are conducted to determine the acceptability or unacceptability of risk to the organization and how to manage the risk. When the assessed risk exceeds the organization's acceptable range, experts should offer methods and strategies to reduce it to a manageable level. Implementing these measures is expected to achieve the desired level of safety within the organization [8].

The challenge of selecting from the proposed control actions to address risk is prominent in this area. Numerous risk assessment strategies need to offer a systematic approach for assessing the effectiveness or cost-effectiveness of control measures, thus relying on experts to make the final decisions. This situation creates a void, as the lack of a structured evaluation mechanism leaves a fundamental step in risk management unresolved. Opting for the appropriate control measure or measures constitutes a decision-making predicament, and its accurate resolution holds paramount significance in risk management [9,10]. In general, the fundamental objective of risk analysis and assessment is to determine the degree of uncertainty within the system under examination, along with its associated costs, and suggest appropriate solutions to mitigate risks and minimize expenses [11].

Among the various methods employed for risk assessment, the William Fine approach stands out for its incorporation of an indicator to evaluate the efficacy of control measures. The Frank and Morgan risk assessment method also estimates the economic cost. However, this method is more used for prioritizing units to allocate financial and human resources [12].

The William Fine method, introduced by the distinguished William T. Fine, the Head of the Safety Department at the Naval Warfare Laboratory in Maryland, USA, in 1971, has been widely acknowledged and implemented in the domain. This approach uniquely incorporates an economic analysis of safety measures, providing a rationale for the costs involved in addressing risks. By doing so, it enhances the overall effectiveness of risk assessment. Unlike many other risk assessment methods, the William Fine method offers a clear economic justification for safety interventions [13]. Furthermore, studies indicate that since its inception in 1971, the William Fine method has employed cost tables to determine the justifiability of corrective actions, with values denominated in dollars for various industries. However, notably, the economic capability of different industries to finance corrective actions varies across countries [14,15].

Since implementing safety interventions necessitates financial expenditures and allocating funds for such initiatives constitutes a fraction of the organization's overall budget, it is imperative to investigate the cost of safety interventions in each industry relative to its economic power [16]. Consequently, the relevant industry can elucidate the J index proficiently, and tailoring the cost invoice table to reflect the economic realities of each industrial sector and employing it to evaluate the risk in Iran is crucial. Furthermore, it is essential to revise the calculation tables of the aforementioned index for a variety of sectors within the Iranian industry by taking into account economic indicators to enable researchers and managers to utilize it.

This study intends to take a fresh look at the J index of the William Fine method and use its power to assist in decision-making to select control strategies. The William Fine method appears to have been overlooked, and consequently, the potential of the J index remains untapped due to outdated cost invoice tables. Accordingly, this study addresses this by updating the “cost factor” of the William Fine method to reflect the current economic conditions in selected industrial manufacturing sectors in Iran.

## 2 Literature review

### 2.1 Application of William fine method in risk assessment in different industries

Preventing accidents necessitates an initial investigation and assessment of their underlying causes, which is crucial for avoiding the repetition of comparable incidents [17]. Risk assessment is one of the methods of risk control in the industry. The analysis of studies pertaining to the William Fine risk assessment method demonstrates that this approach is primarily employed for risk rating. Notably, only a tiny fraction of the studies have addressed the calculation of the justifiability index for control measures. In 2018, Carpinteiro et al. used the William Fine method to analyze the risks during the execution phase in the Aguas Santas tunnel. Risks were identified for each activity. Control measures were suggested for dangerous risks [18].

In 2019, Colim et al. used the William Fine method with the aim of evaluating the risks in different workstations by robots and human interactions in a large furniture manufacturing company. Twenty workstations were evaluated, four of which were related to human control of robots. In this study, the William Fine method was used only to rank the risks, and the J index was not calculated to justify the cost of corrective measures [19]. Hafizi et al. (2018) used the William Fine method to identify danger points and assess risk in the acid recovery unit of Abadan Oil Refining Company and calculated the cost justification index [15]. In 2023, Mohebi et al. carried out an environmental risk assessment in the mountain and recreation area of Derke, Tehran, Iran, using the William Fine method [20]. Moreover, Nasari Ardakani et al. conducted an environmental risk assessment using the combined PHA and William Fine methods in the Eram Ardakan tile and ceramic factory in 2021. However, they did not calculate the J index [21].

### 2.2 Costing of accidents and investments in safety

Research indicates that assessing the cost of accidents in industries is challenging. In 2016, Alonso et al. introduced a classification system aimed at managing safety costs on construction sites. Their study revealed a lack of independent financial data regarding preventive costs. Finally, they presented a proposed classification for health and safety costs in construction sites [11]. In 2018, Atehnjia et al. conducted a study with the aim of providing a model for accident analysis in order to develop control safety measures based on the cost-effectiveness approach to prevent accidents in dry docking and undocking operations. They used a cost-effectiveness approach to compare the costs of implementing these measures against their effectiveness in preventing accidents. In this context, cost refers to the financial investment needed, while the reduction in the number of fatalities per year measures effectiveness [22].

In 2018, Totunchian et al. designed a parametric model to identify the parameters governing safety costs in oil and gas projects. The parameters related to the safety management system in oil and gas projects were extracted by examining the contracts related to these industries and related standards. They reached a final checklist related to safety management system parameters using the Delphi method. Using the conceptual model, they calculated the total cost of the project until 2015. Notably, the safety management determined costs for different project stages and the relevant weighting factors separately. The results showed that the weight factor of safety management is 1.1 % of the total weight factor of the project [23].

### 2.3 Application of the Delphi method in safety studies

Given that the direct cost of health and safety measures in industries is often not readily available, the Delphi method offers a collaborative approach to estimating these costs. This method involves a multi-stage process of gathering expert opinions through a completed questionnaire with individual feedback, ultimately aiming to reach a consensus. The iterative nature of the Delphi method, with results being polled in several stages, enhances the accuracy of the estimates. For instance, in 2017, Ardeshir et al. successfully used the Delphi method with the fuzzy AHP approach to identify and prioritize factors influencing the construction industry [24].

Moulaifar et al. (2017) used the Delphi method, Fuzzy Hierarchical Combined Method (FAHP), and TOPSIS to identify and prioritize control strategies for facing heat stress in the rubber industry. Their study showed that using the Delphi method, the crucial criteria and strategies for controlling heat stress can be identified and screened in the rubber industry [25]. In 2016, Ramos et al. employed the Delphi method to explore the impact of various factors on occupational health and safety assessments. Their study concluded that transferring risks and implementing preventive measures can be financially feasible for companies and beneficial for society. This is achieved through public initiatives like taxation, penalties, and regulatory laws. They emphasized that the Delphi method is a crucial tool in analyzing occupational health and safety [26].

## 3 Material and methods

The present study is applied, focusing on evaluating the William Fine risk assessment method. This method centers on calculating and assessing risk score (RS) through a specific equation (1).

Equation (1) RS=C×E×P

In this context, the factors of consequence intensity (C), exposure rate (E), risk probability (P), and RS are of significant importance. A group of experts is responsible for assigning a score to each of the aforementioned factors, grounded on the present circumstances, utilizing the three fundamental tables presented in this methodology. Planning, anticipating, and estimating corrective action when a hazard is identified is imperative. Subsequently, upon determining the RS, the calculation of acceptable costs is derived from Equation (2), whereby J denotes the cost justification index, CF represents the cost factor, and DC signifies the degree of correction. These numerical values of DC and CF are presented in the standard tables of the methodology (Table 1, Table 2).

**Table 1:** Cost factor.

Definition of criteria (the expenditure required to eliminate and attenuate hazards)	CF Factor
Over $ 50,000	10
$ 50,000 - $ 25,000	6
$ 25,000 - $ 10,000	4
$ 10,000 - $ 1,000	3
$ 1000 - $ 100	2
$ 100 - $ 25	1
Under $ 25	0.5

**Table 2:** Degree of correction (DC).

Definition of criteria	DC Factor
The complete elimination of risk is a certainty, with a probability of 100 %	1
At a minimum of 75 %, the risk is mitigated	2
Between 75 and 50 percent of the associated risk has been mitigated	3
Between 50 and 25 percent of the potential hazard is effectively mitigated	4
Less than a quarter of the potential hazard is mitigated	6

The cost factor pertains to the scale of cost estimates for the proposed corrective actions. The correction level indicates the degree of corrective action proposed to mitigate or curtail the risk and to avert or terminate it. Estimation is grounded on expertise and knowledge associated with the relevant activity. According to the William Fine approach, the acceptability of control costs or risk elimination is contingent upon the value of J, whereby such measures are deemed acceptable when J is greater than or equal to 10 and unacceptable when J is less than 10 [13].

Equation (2) J=RS/(DC×CF)

### 3.1 Selection of Delphi panel members

Due to the impossibility of accurate access to cost data and safety investment in selected industries, the Delphi method was used in this study. For this purpose, the opinions and judgments of individuals are collected in a specific area. In other words, judgment is left to experts [27,28].

Limited access to financial security information stems from several factors. Firstly, industrial companies vary in their definitions of safety-related expenses, including costs for safety training, incentives, personnel, and equipment. Additionally, in the Iranian economic system, information about the financial costs associated with safety was not publicized. After providing sufficient explanations about the project's objectives and obtaining informed consent, the subjects entered the study, and the questionnaires were provided. Therefore, thirty experts in the field of occupational health and safety participated in this Delphi study.

**Fig. 1:**
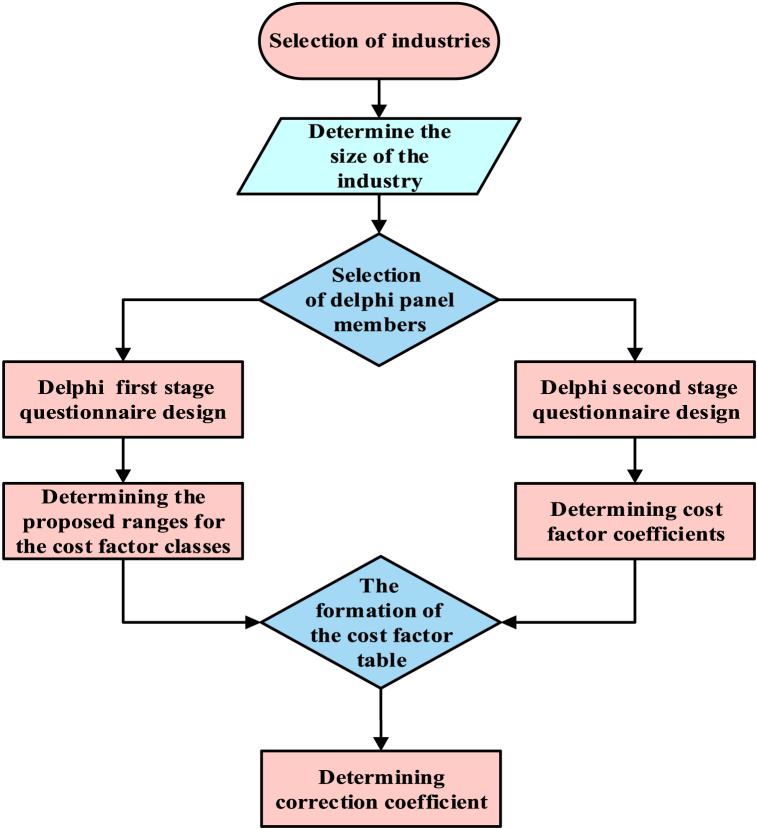
Diagram of the study implementation steps.

The criterion for entering the study was working experience of at least one year in the desired industry. To complete the questionnaire, participants must currently be employed in the relevant industry and hold a certificate in occupational health, industrial safety, or HSE. Fig. 1 illustrates the steps involved in implementing the study and provides detailed descriptions of each step below.

### 3.2 Selection of industries

Selecting industries was based on the International Standard Industrial Classification (ISIC) System. The ISIC Classification is an international reference classification of manufacturing activities. Industry selection was based on the International Standard Industrial Classification (ISIC) System, an international standard for categorizing manufacturing activities. From section C, which covers industrial production, the selected divisions are food products (division 10), drugs and pharmaceuticals, chemical and herbal products (division 21), and the automotive industry (division 29). These divisions were chosen for their accessibility.

The division responsible for the production of food items has identified a category encompassing various other food products, including the manufacturing of bakery goods, confectioneries, biscuits, sugar, cocoa, chocolate, and sugary treats. In the division dedicated to producing medicines and pharmaceuticals, a selection has been made of chemical and herbal products categorized under the same nomenclature. Additionally, the automotive division has focused on the group and classification pertaining to the production of vehicles, as well as automotive parts and accessories. Selecting the production divisions from Section C included large and small industries, as well as the most critical industries directly related to the behavior and livelihood of the Iranian people.

Production divisions selected were based on the ISIC code, and the sampling method available in each category was used. The two-digit ISIC codes were considered as divisions, and within each division, several classes were selected (ten workshops from each division based on the initial evaluation and the level of expert participation). When a class was selected, it was considered a selection criterion. For example, industries with the slightest difference in nature and the most homogeneity and similarity within the class were selected. Additionally, having access to workshops was a key factor in the selection process.

### 3.3 Determine the size of the industry

The characterization of small and medium enterprises in Iran is subject to variation across different organizations. These organs and organizations, each with their unique work requirements, have played a crucial role in the classification process. For instance, the Statistics Office of the Central Bank of Iran, has considered classifying industrial units (based on the number of employees), as listed in Table 3 [29]. This collaborative effort has resulted in a comprehensive understanding of the industry sizes, with each selected industry being divided into four categories based on the population employed: little, small, medium, and large.

**Table 3:** The classification of industrial units based on the Statistics Office of the Central Bank of Iran.

Number of Employees	Classification
Less than 10 employees	Little
10 to 49 employees	Small
50 to 99 employees	Medium
More than 100 employees	Large

### 3.4 Questionnaire design and the implementation of the first stage of the Delphi technique to determine safety cost ranges

By studying the research background, cases related to the cost of safety interventions in the industry were extracted. Accordingly, the present study aimed to clarify the concept of safety interventions for relevant experts. The initial questionnaire was prepared and distributed to a group of experts. Before sending out the survey, participants received a detailed explanation of the questionnaire's specifics, including its overall content and the study's objectives. The questionnaire was sent to 30 experts, with ten individuals representing each production division. It comprised sections that covered the profiles of the respondent experts, examples illustrating safety interventions, and the core research questions. Aligned with the study's objectives, two key questions were posed: the company's total expenses during the year and the safety-related expenditures within the industry. The results from this questionnaire revealed a range of minimum and maximum safety costs for each production division.

### 3.5 Determining the proposed ranges for the “cost factor” classes

For each of the production divisions, based on statistical information, the relationship between the rows of the main table, applying a series of coefficients, and based on the research team's opinions, the proposed ranges for the classes of the cost factor table were presented. Based on the results of the Delphi technique in the previous stage and statistical information, seven proposed ranges were determined, which were replaced in the first column of the cost factor table. In the cost factor table, the maximum safety cost was categorized as the highest level, while the minimum safety cost was categorized as the lowest level. The values between these two are placed in the intermediate rows of the table.

### 3.6 Questionnaire design and implementation of the second stage of the Delphi technique to achieve “cost factor” coefficients

In William Fine's cost factor table, each cost interval is represented by a rating number (CF coefficient) representing the cost factor. These numbers are weighted and lack distinct intervals, so they were chosen using a fuzzy logic method, which is the preferred approach. This process was applied individually to each production sector. A fuzzy set is a collection where membership boundaries are not clearly defined. This theory employs ambiguous terms instead of precise numbers [30].

The second stage of the Delphi questionnaire was prepared based on the data from the first stage. This study aimed to explore two key questions: How many employees are involved, and what do experts think about the different cost ranges in the cost factor table? During this phase, experts were asked to share their perceptions of the proposed cost periods concerning safety investments within their companies. Additionally, they provided insights into how management allocates these cost ranges for safety expenditures and their willingness to approve and pay for them. Table 4 was used in the language variables questionnaire [31]. Individuals were asked to select an item (very high, high, and very low) on the Likert scale for each suggested interval.

**Table 4:** Linguistic expressions and fuzzy Delphi numbers.

Triangular fuzzy numbers	Linguistic expressions
(0, 0, 0.25)	Very low
(0, 0.25, 0.5)	Low
(0.25, 0.5, 0.75)	Medium
(0.5, 0.75, 1)	High
(0.75, 1, 1)	Very high

In fuzzy logic theory, the types of memberships can be drawn according to the intended application. However, two types of membership functions are used widely: Trapezoidal and triangular [30]. This research used the membership function of triangular fuzzy numbers [32]. Screening results are obtained by comparing each index's acquired value with a predetermined threshold. The decision-maker's judgment influences this threshold and directly impacts the number of factors being screened. Establishing a threshold is not straightforward or universally regulated. In this study, the value of 0.7 was considered the threshold value. The triangular fuzzy values concerning expert opinions were initially computed. The fuzzy average was then determined to establish the average of the respondent's views. The value of the fuzzy number τ was calculated for each of the indices using the following equations [32].

Equation (3) $${\tilde{\tau }}_{\text{ij}}=\left(a_{\text{ij}},b_{\text{ij}},c_{\text{ij}}\right),i=1,2,\ldots ,n\;j=1,2,\ldots ,m$$

Equation (4) $$a_{j}=\sum \frac{a_{\text{ij}}}{n}$$

Equation (5) $$b_{j}=\sum \frac{b_{\text{ij}}}{n}$$

Equation (6) $$c_{j}=\sum \frac{c_{\text{ij}}}{n}$$

In the above equations, the index denoted as ‘i' pertains to the expert, while the index represented as ‘j' pertains to the decision index. Moreover, the fuzzy ransom value of the average fuzzy number was computed using the following equation [30].

Equation (7) $$\text{Crisp}=\frac{a+b+c}{3}$$

The initial step involved transforming the data into a fuzzy number, as indicated by the spectrum in Table 4. Subsequently, utilizing Equations Equation (4), Equation (5), Equation (6)), the fuzzy mean was derived from the scores. Finally, through Equation (7), this fuzzy mean was converted into a precise numerical value. Accordingly, the intervals with a definite score between 0.7 and 1 had the most emphasis from the respondents' point of view. Indicatively, the definite score between 0.5 and 0.7 had a medium emphasis, and less than 0.5 had a low emphasis. Using the results of the second Delphi stage and the main cost factor table, the William Fine method was applied to the numerical values of the cost factor coefficient.

### 3.7 Determine the correction coefficient for the cost factor table

Due to exchange rate fluctuations and the possibility of using the cost factor table over time, the Producer Price Index (PPI) was examined, and the effect of this index was discussed on the cost invoice table. According to international standards, the PPI measures the price change of goods and services bought by manufacturers and sold as final products. It serves as an early indicator to predict inflation trends [33]. Thus, the numerical value of the PPI index in the last five years was extracted from the Central Bank's website. The changes in this index over the past five years were separately analyzed for the food, pharmaceutical, and automotive industries. The average growth rate for each sector was calculated and projected for future years.

## 4 Results

This study collected safety cost information from three divisions: Automotive, pharmaceutical, and food, based on access to occupational safety and health experts. The size of the industries was determined based on the number of employees presented in Table 5. Accordingly, the automotive and pharmaceutical sectors were categorized as major, while the food industries were designated as minor.

**Table 5:** Size of selected industries.

Industry Group						Person code
Food		Pharmaceutical		Automotive		
Industry size	Number of employees	Industry size	Number of employees	Industry size	Number of employees	
Large	More than 100 employees	Large	More than 100 employees	Large	More than 100 employees	1
Small	10 to 49 employees	Small	10 to 49 employees	Large	More than 100 employees	2
Small	10 to 49 employees	Large	More than 100 employees	Medium	50 to 99 employees	3
Large	More than 100 employees	Large	More than 100 employees	Large	More than 100 employees	4
Small	10 to 49 employees	Large	More than 100 employees	Large	More than 100 employees	5
Small	10 to 49 employees	Large	More than 100 employees	Large	More than 100 employees	6
Medium	50 to 99 employees	Large	More than 100 employees	Large	More than 100 employees	7
Small	10 to 49 employees	Large	More than 100 employees	Medium	50 to 99 employees	8
Small	10 to 49 employees	Large	More than 100 employees	Large	More than 100 employees	9
Small	10 to 49 employees	Large	More than 100 employees	Large	More than 100 employees	10

The study utilized the Delphi technique, conducted in two stages. Table 6 illustrates the frequency distribution of participants' fields of study, work experience, and educational degrees. The majority of respondents held degrees in occupational health. In Iran, occupational health graduates typically manage safety and health responsibilities within the industry. The average work experience of the respondents was 11.9 in the automotive group, 6.7 in the pharmaceutical group, and 6.1 in the food industry group.

**Table 6:** Frequency distribution of participants in the Delphi technique.

Frequency (%)	Frequency (%)	Frequency (%)	Frequency (%)	Frequency (%)	Frequency distribution
Crisis management	**Ergonomics**	**HSE**	**Industrial safety**	**Occupational health**	**Field of study**
0 (0)	1 (10)	1 (10)	1 (10)	7 (70)	Automotive
0 (0)	0 (0)	1 (10)	1 (10)	8 (80)	Pharmaceutical
1 (10)	0 (0)	2 (20)	1 (10)	6 (60)	Food

15 years and older		**11**–**15 years**	**6**–**10 years**	**1**–**5 years**	**Work experience**

2 (20)		3 (30)	4 (40)	1 (10)	Automotive
0 (0)		1 (10)	5 (50)	4 (40)	Pharmaceutical
0 (0)		2 (20)	3 (30)	5 (50)	Food

	**Masters**		**Bachelor**		**Degree of education**

5 (50)			5 (50)		Automotive
5 (50)			5 (50)		Pharmaceutical
4 (40)			6 (60)		Food

### 4.1 Results of the first stage of the Delphi technique

The first stage questionnaire was collected. Since the present study aimed to obtain the standard cost factor table, the minimum and maximum cost range expected by experts with some minor changes (to achieve a random number) were used. The minimum and maximum cost ranges are listed for each industry in Table 7:

**Table 7:** Results of the initial stage of the Delphi technique (values in a million Rials).

Industry Group						Person code
Food		Pharmaceutical		Automotive		
Expected cost	Cost spent	Expected cost	Cost spent	Expected cost	Cost spent	
1360	960	10000	10000	58875	58875	1
400	350	660	425	2000	250	2
1000	100	5000	4000	2200	1200	3
1500	1000	15000	9527	10000	8000	4
1200	940	4000	3200	3500	3000	5
1000	600	1416	1140	50000	30000	6
2000	1200	500	300	3500	2500	7
1000	300	900	802	2000	1365	8
1400	800	2000	1100	4000	2810	9
1450	1400	4200	3000	34000	23000	10
400		**500**		**2000**		Minimum cost
2000		**15000**		**60000**		Maximum cost

**Fig. 2:**
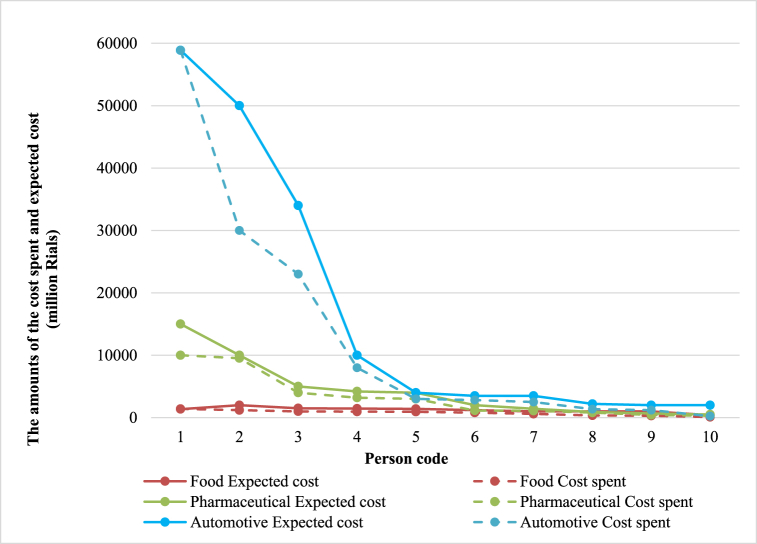
Comparison of cost amounts spent and expected cost in different industries based on the experts' views.

Fig. 2 compares the amounts spent and expected costs in the automotive, pharmaceutical, and food industries based on the experts' views. As can be seen, the difference between the minimum and maximum costs in the automotive industry compared to the pharmaceutical and food industries is gigantic because both small and huge automotive industries vary significantly in the amount of money they spend on safety.

**Fig. 3:**
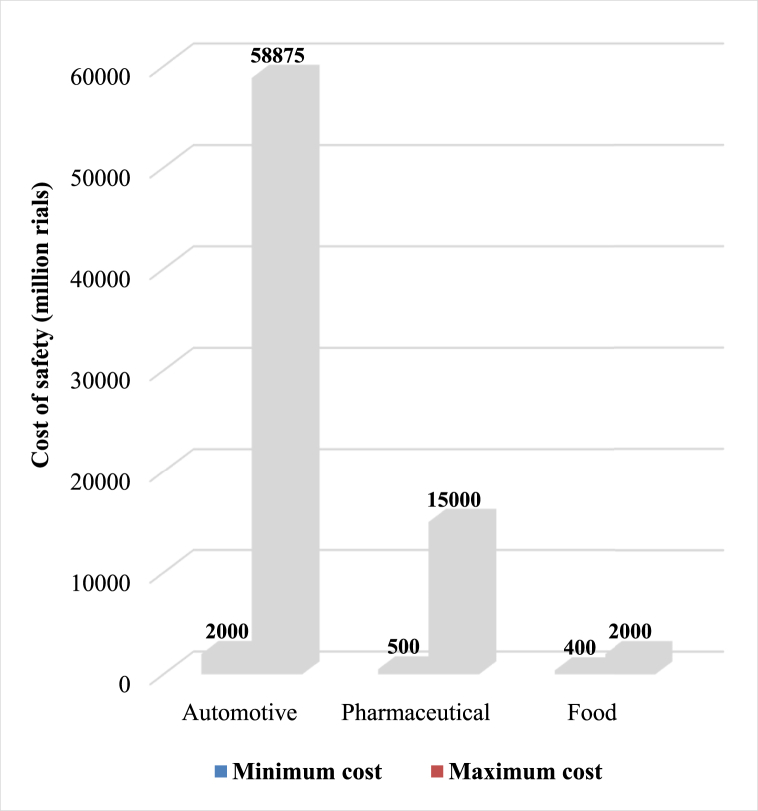
Comparison of the minimum and maximum cost ranges of the automotive, pharmaceutical, and food industries.

Fig. 3 illustrates the minimum and maximum cost ranges for the automotive, pharmaceutical, and food industries. Notably, the highest safety cost in the food industry, 200 million Rials, is equivalent to the lowest safety cost in the automotive industry. This highlights the disparity between these two industries, underscoring their differences in scale regarding annual production and revenue.

### 4.2 Provide proposed ranges for the categories of the “cost factor” table for each selected economic sector

Seven proposed ranges, replaced in the first column of the cost factor table (Table 8), were determined from the first stage results of the Delphi technique based on statistical information.

**Table 8:** Proposed cost ranges for each industry (in a million Rials).

Food	Pharmaceutical	Automotive	Industry Group
More than 2000	More than 15,000	More than 60,000	Suggested cost ranges
2000–1700	15000–12000	60000–40000	
1700–1300	12000–7000	40000–30000	
1300–800	7000–2000	30000–10000	
800–500	2000–700	10000–3000	
500–400	700–500	3000–2000	
Less than 400	Less than 500	Less than 2000	

### 4.3 Presentation of CF coefficient related to classes of “cost factor” table

The CF coefficient of the cost factor table was determined using the Delphi second-stage questionnaire results for each automotive, pharmaceutical, and food industry. Table 9 gives the importance of each proposed cost interval in the cost factor table from the point of view of the expert group and the results of all fuzzy calculations in the first Delphi phase in the three industries of automotive, pharmaceutical, and food.

**Table 9:** Results of experts' view and fuzzy Delphi of the automotive, pharmaceutical, and food industries.

Proposed cost factor coefficient	Suggested range (in million Rials)	Fuzzy Delphi results			Results Expert Comments					Row	Industry Group
		Condition	Definitive score	Fuzzy points	Significance						
					Very much	Much	Medium	Low	Very low		
10	More than 60,000	Most emphasis	0.917	(0.75,1,1)	10	0	0	0	0	1	Automotive
7	60000–40000	Most emphasis	0.867	(0.68,0.93,1)	7	3	0	0	0	2	
5	40000–30000	Most emphasis	0.817	(0.63,0.88,0.95)	7	1	2	0	0	3	
3.5	30000–10000	Most emphasis	0.733	(0.53,0.78,0.9)	5	2	2	1	0	4	
2	10000–3000	Medium emphasis	0.525	(0.3,0.53,0.75)	1	2	5	1	1	5	
1	3000–2000	Low emphasis	0.283	(0.13,0.25,0.48)	1	0	2	2	5	6	
0.5	Less than 2000	Low emphasis	0.183	(0.05,0.13,0.38)	0	1	0	2	7	7	
10	More than 15,000	Most emphasis	0.883	(0.7,0.95,1)	8	2	0	0	0	1	Pharmaceutical
7	15000–12000	Most emphasis	0.833	(0.65,0.9,0.95)	8	0	2	0	0	2	
4.5	12000–7000	Most emphasis	0.792	(0.6,0.85,0.93)	7	1	1	1	0	3	
2.5	7000–2000	Medium emphasis	0.533	(0.3,0.55,0.75)	2	0	6	2	0	4	
1.5	2000–700	Low emphasis	0.292	(0.08,0.28,0.53)	0	1	1	6	2	5	
1	700–500	Low emphasis	0.158	(0.03,0.1,0.35)	0	0	1	2	7	6	
0.5	Less than 500	Low emphasis	0.100	(0.0,0.03,0.28)	0	0	0	1	9	7	
10	More than 2000	Most emphasis	0.842	(0.65,0.9,0.98)	7	2	1	0	0	1	Food
7	2000–1700	Most emphasis	0.817	(0.63,0.88,0.95)	7	1	2	0	0	2	
4.5	1700–1300	Most emphasis	0.700	(0.5,0.75,0.85)	6	0	2	2	0	3	
2	1300–800	Low emphasis	0.425	(0.18,0.43,0.68)	0	1	5	4	0	4	
1.5	800–500	Low emphasis	0.308	(0.08,0.3,0.55)	0	1	1	7	1	5	
1	500–400	Low emphasis	0.158	(0.03,0.1,0.35)	0	0	1	2	7	6	
0.5	Less than 400	Low emphasis	0.125	(0.03,0.05,0.3)	0	0	1	0	9	7	

### 4.4 Determine the correction coefficient for the cost factor table

Based on the official data published by the Central Bank of Iran, the PPI's numerical values were extracted from 2012 to 2017, and the time series of changes in this index were separately calculated for each food, pharmaceutical, and automotive industry in these five years. Furthermore, each year's growth rate compared to the previous year was calculated through Equation (8). The average growth rate of five years was calculated. Thus, the average growth rate of five years was obtained and generalized to future years (Table 10).

Equation (8) $$r=\frac{\;X_{t}-X_{t-1}}{X_{t-1}}$$

**Table 10:** Numerical values of the PPI index and calculation of average growth rate.

Automotive		Pharmaceutical		Food		time series
Growth rate	PPI index	Growth rate	PPI index	Growth rate	PPI index	
	127.3		117.2		142.4	2012
43 %	182.1	66.6 %	193.3	34.6 %	191.6	2013
17.6 %	214.2	17.3 %	229.1	13.3 %	217.1	2014
5.6 %	226.3	8.9 %	249.6	8.8 %	236.2	2015
2 %	230.8	11.9 %	279.3	5.9 %	250.2	2016
3.8 %	239.5	6 %	296.1	7.4 %	268.8	2017
14.4 %		22.2 %		14.1 %		Average growth rate
1.144 %		**1.222** **%**		**1.141** **%**		**correction coefficient of the cost factor table**

The value of r is the growth rate, X_t_ is the numerical value of the PPI index in year t, and X_t-1_ is the numerical value of the PPI index in year t-1.

The values of the correction coefficient for the coming years will be generalized based on the average growth rate calculated for each industry. For instance, in the food industry, the figures from 2017 are multiplied by 1.141 for 2018. Similarly, the figures from 2018 are multiplied by 1.141 for 2019, and this pattern continues for 2020, where the 2019 figures are multiplied by 1.141. This method will be consistently applied in future years.

## 5 Discussion

One of the main factors in health and safety management is developing and implementing risk assessment, analyzing an organization's status to ensure the success of health and safety programs [8]. Workplace safety measures can reduce the likelihood of accidents and the organization's liability for accidents.

One of the crucial issues in the safety management process is the economic valuation of safety controls to decide on the appropriate and optimal control solution. A significant challenge we face in this area is the absence of an adequate tool for evaluating various control methods and determining the most appropriate control solution from an economic perspective. Safety experts and people conducting risk assessments need a reliable indicator to select control strategies. William Fine's method helps managers make better economic decisions by providing an index for assessing the effectiveness of control measures. Therefore, updating the cost factor table in the J Index of the William Fine method, tailored to the type of industry, helps them have a reliable approach. Updating this table makes it possible to achieve a more accurate estimate of control costs or risk elimination for selected industries.

Alternative approaches, such as the Frank and Morgan method, exist for conducting risk assessments and estimating economic costs. This methodology assists managers in determining the necessary capital investments required to achieve optimal risk reduction across various factory units and facilitates comparisons of risk levels among different factories. In contrast, while the Frank and Morgan method primarily focuses on prioritizing units for allocating financial and human resources, the William Fine risk assessment method comprehensively estimates the costs associated with risk control for all identified high-risk hazards. Additionally, it is more straightforward and less complex than the Frank and Morgan method, making it a viable option for experts across various industries seeking a rapid evaluation of control measures [34].

This study revealed that the economic strength of different industries varies in terms of their safety expenditure. The food industry, in particular, is identified as having less financial power than the pharmaceutical and automotive industries and is classified as a smaller sector. When comparing safety budgets, the food industry spends considerably less annually than its pharmaceutical and automotive counterparts. This discrepancy can be traced back to the industry's size, as safety spending is influenced by production costs and the revenue generated each year, establishing a link between these financial elements. The cost factor coefficient is located in the denominator in the cost justification index formula. This means that the value of the CF coefficient significantly impacts the J index. Specifically, a higher CF coefficient results in a lower J index, and a lower CF coefficient results in a higher J index. Additionally, because financial and monetary values vary across industries, the assigned coefficients for each cost category in the cost factor table will differ depending on the industry. For example, in the current study, the value of the CF coefficient for the fourth row of the proposed cost range in the automotive industry (10,000 to 30,000 million Rials) is 3.5, and in the food industry (1,700 to 1,300 million Rials) is 2. At the same time, the value of the CF coefficient for the fourth row of the cost range in the main table ($1000 to $10000) is assigned the number 3 for all industries. Therefore, considering that the cost factor table has been updated, taking into account the difference in the financial value of different industries, experts can use this table in periodic evaluations to find an economically justified control solution to reduce risks.

Company size can also represent a competitive advantage. Since a larger market share requires more production and sales, having sufficient financial resources and a larger size can help the company produce more to create competitive advantages. In 2019, Nalarreason et al. conducted a study to investigate the effect of company size on earnings management. Their study was based on data from 2013 to 2017, focusing on manufacturing companies listed on the Indonesia Stock Exchange. They concluded that company size positively and significantly affects the profit management of manufacturing companies [35].

In 2013, Feng examined the impact of safety investments on the safety performance of construction projects. Their research showed that safety performance and investments are not independent of the size of companies or projects and are interrelated. Generally, larger companies or projects are associated with a higher safety culture and a higher project risk level [36].

This study's findings reveal a significant disparity between the minimum and maximum costs in the automotive industry compared to the pharmaceutical and food industries. Specifically, the highest safety costs in the food industry, amounting to 2000 million Rials, align with the lowest safety costs in the automotive industry. Due to the difficulty of directly accessing industry safety costs, the Delphi method was employed to determine the range of minimum and maximum costs. Numerous studies have explored occupational accidents and health and safety expenses. In a thorough review of 29 studies, Jallon et al. discovered the direct and indirect expenses associated with occupational mishaps. Among these studies, nine employed a top-down methodology with national data and statistics to determine the average cost randomly. Seven studies utilized a bottom-up approach, where the average cost was determined through surveys and interviews. The remaining 13 studies were founded on data gathered at the level of individual firms, with seven conducting some cost-benefit analysis [37].

In their 2016 study, Alonso et al. introduced a classification framework aimed at managing safety costs in construction environments. They collected cost-related data through a questionnaire. The study concluded that construction site accounting departments fail to identify safety and health costs. Based on their findings, they also calculated the costs associated with accident prevention. Notably, the research highlighted the absence of independent financial information pertaining to preventative costs [11]. The current study used a questionnaire to achieve safety costs due to the lack of direct access to financial data.

In 2018, Tehrani et al. scrutinized the influence of safety investment expenses on the safety culture observed in construction industry projects. This research aims to explore the interdependent impacts of safety investment components, encompassing the costs of safety training, safety disruptions, safety personnel, and safety equipment, on safety culture in power plant construction projects in Iran. For this purpose, an approach was used based on structural equation modeling. In addition, the results indicated that the proposed model for measuring safety culture has a good fit, and in general, spending costs to invest in safety improves safety culture in projects and thus reduces work accidents [38].

One of the limitations of this study was the absence of direct access to the financial data of the selected industries, coupled with the reliance on statistics from industrial workshops available on the website of the Statistics Center of Iran. Additionally, there was a lack of access to precise figures regarding safety costs within the industries, constraints related to the insufficient number of industries, particularly in the automotive sector, and the considerable workload faced by the specialists and experts involved in the study.

## 6 Conclusion

When considering safety measures and their costs in industrial design, many in the field tend to focus on essential, low-cost items like helmets and gloves. However, only a few consider the long-term safety and security principles in industrial design and the significant impact these can have on profitability. Despite human challenges and financial setbacks, this critical issue incurs significant costs that may jeopardize a project's profitability if long-term safety concerns are not addressed. Safety costs seem to increase the costs of all industries. Accident statistics indicate that investing in safety measures is the only cost that can actually increase profits and revenues for industrial facilities. When workplace accidents occur due to non-compliance with safety standards, organizations face a range of expenses. These include medical bills for the injured, downtime during the incident, paid leave for injured employees, overtime pay for others covering the injured worker's tasks, fines, and compensations. Additionally, the impact on the injured employee is significant. In high-risk work environments, failing to comply with safety protocols and not investing adequately in safety can lead to substantial costs for the organization.

Industrial managers often need more awareness about the costs involved in implementing workplace safety measures, which can hinder the effective execution of these programs. This study was initiated with no existing research on the economic aspects of safety controls and without economic tools to assess the costs of safety interventions in industries. This research is crucial for bridging this gap and highlights the need to explore this issue further in future studies. If a company's management acknowledges the significance of safety and its implications, as well as comprehends the impact of safety expenditures and safety initiatives on the organization's financial framework, it will seek specialized programs to oversee its safety-related costs. Achieving this requires a clear understanding of the safety framework and cost trends. The present study assists occupational health and safety professionals in the industry in persuading managers to allocate resources toward safety initiatives. Undoubtedly, it can help Iran's industrial managers better understand risk control costs and their effects on long-term industry profitability and make better decisions.

This research determined safety cost ranges in selected manufacturing industries using a Delphi questionnaire and expert consultations. Future studies should consider employing additional methods and tools to achieve more precise data. Moreover, assessing how closely the safety cost ranges obtained in this study reflect reality is essential. There is also a recommendation to extensively adapt the cost factor table for other industrial production and economic sectors within Iran's industries. The findings from this research have been used to compare safety costs across different industries and evaluate the acceptability of these costs concerning identified risks in these industries.

## CRediT authorship contribution statement

**Bafrin Moloudpourfard:** Writing – review & editing, Writing – original draft, Methodology, Investigation, Formal analysis, Conceptualization. **Morteza Tahamipour Zarandi:** Writing – review & editing, Writing – original draft, Supervision, Methodology, Conceptualization. **Mostafa Pouyakian:** Writing – review & editing, Writing – original draft, Supervision, Methodology, Investigation, Formal analysis, Conceptualization.

## Data availability statement

The authors confirm that the data supporting the findings of this study are available within the article.

## Ethics declaration

This study was reviewed and approved by the Research Ethics Committees of School of Public Health & Neuroscience Research Center - Shahid Beheshti University of Medical Sciences with the approval number: IR.SBMU.PHNS.REC.1398.063, dated 2019/07/23. All participants provided written informed consent to participate in the study and for their data to be published.

## Funding sources

This research did not receive any specific grant from funding agencies in the public, commercial, or not-for-profit sectors.

## Declaration of competing interest

The authors declare that they have no known competing financial interests or personal relationships that could have appeared to influence the work reported in this paper.
